# Structure and Magnetic Properties of the *n* = 3 Ruddlesden–Popper Oxyfluoride La_0.5_Sr_3.5_Fe_3_O_7.5_F_2.6_

**DOI:** 10.1021/acs.inorgchem.4c02835

**Published:** 2024-10-18

**Authors:** Andy Bivour, Jonas Jacobs, Florian Daumann, Gerald Hörner, Birgit Weber, Clemens Ritter, Stefan G. Ebbinghaus

**Affiliations:** † Martin-Luther-University Halle-Wittenberg, Department of Chemistry, Inorganic Chemistry, Kurt-Mothes-Straße 2, D-06120 Halle, Germany; ‡ Friedrich-Schiller-University Jena, IAAC, Humboldtstraße 8, D-07743 Jena, Germany; § Department of Chemistry, University of Bayreuth, D-95447 Bayreuth, Germany; ∥ Institut Laue-Langevin, 71 Avenue des Martyrs, F-38042 Grenoble Cedex 9, France

## Abstract

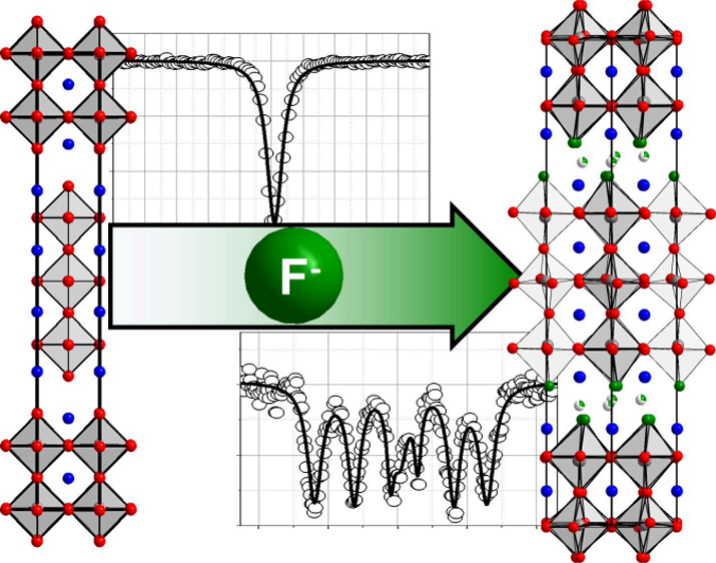

Ruddlesden–Popper (RP) compounds of the general formula (AX)(ABX_3_)_*n*_ with their unique sequence of perovskite-like (ABX_3_) and rock-salt-like units (AX) promise applications in diverse fields such as catalysis and superconductivity. Fluorination of RP oxides often leads to dramatic changes in the material properties, caused by differences in the atomic and electronic structure. While current research focuses on fluorination of *n* = 1 type RP oxides (A_2_BO_4_), *n* = 3 RP oxyfluorides have remained elusive. We present the synthesis of the first iron-based *n* = 3 RP oxyfluoride, La_0.5_Sr_3.5_Fe_3_O_7.5_F_2.6_, which was obtained from an oxide precursor by topochemical fluorination with poly(vinylidene fluoride). Joint Rietveld refinements of neutron and powder X-ray diffraction data were used to determine the crystal structure. Best results were obtained in the space group *Pbca* (No. 61) with *a* = 5.5374(1) Å, *b* = 5.5441(1) Å, and *c* = 29.2541(2) Å. The effect of the aliovalent incorporation of fluoride ions is particularly evident with respect to changes in structure and magnetic properties. The magnetic behavior was studied using field- and temperature-dependent magnetization measurements, Mößbauer spectroscopy, and neutron diffraction. Additional magnetic Bragg reflections observed in the room-temperature neutron data were successfully refined in the space group *Pbca* (61.1.497 in Opechowski–Guccione notation), indicating a G-type antiferromagnetic ordering with a surprisingly high Néel temperature above 300 K. This strong increase of *T*_N_ by several hundred Kelvin compared to the parent oxide is particularly remarkable.

## Abstract 2

Structure and magnetic properties of La_0.5_Sr_3.5_Fe_3_O_7.5_F_2.6_ were investigated by room-temperature X-ray and neutron diffraction, susceptibility measurements, and Mößbauer spectroscopy. As a result, an antiferromagnetic ordering between the iron centers was found to persist above room temperature.

## Introduction

**Figure 1:**
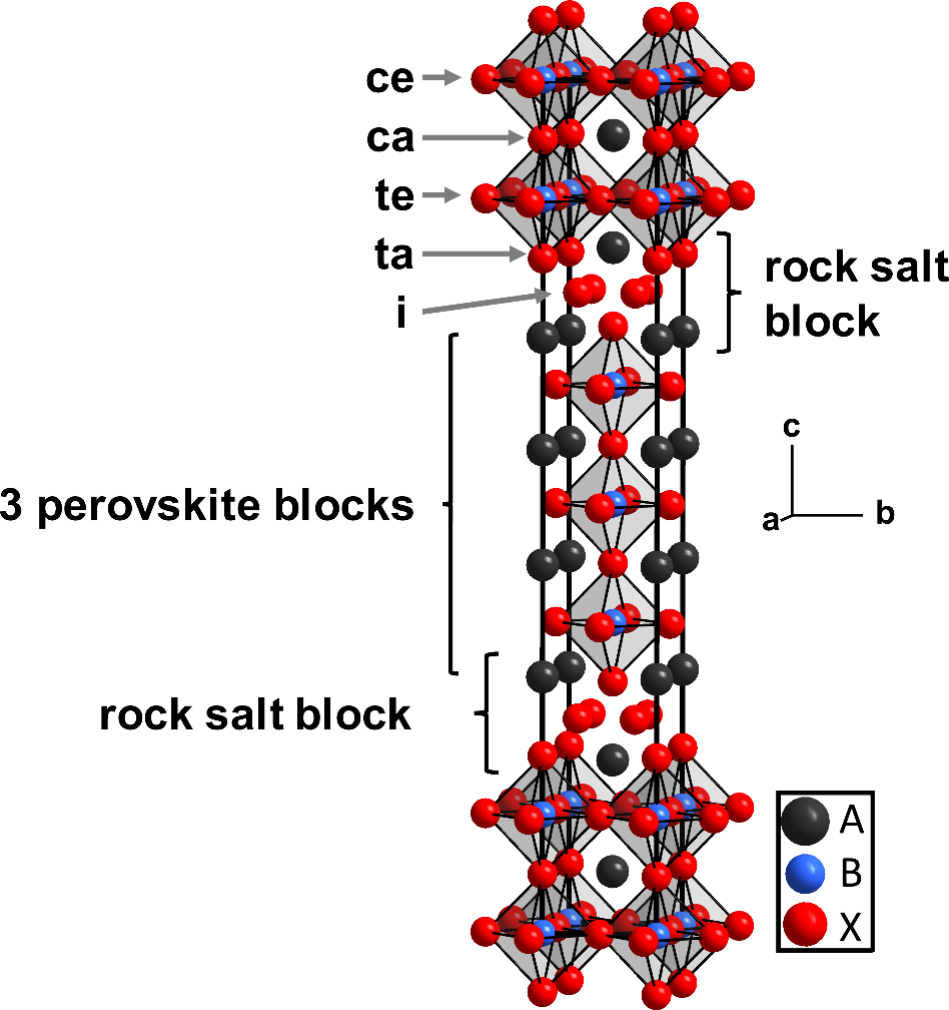
Representation of the *n* = 3 Ruddlesden–Popper structure (space group *I*4/*mmm*). The BX_6_ octahedra are shown, and arrows indicate the five different anionic sites (see text for details).

Compounds of the Ruddlesden–Popper (RP) type possess a layered perovskite structure. Their general formula can be written as (AX)(ABX_3_)_*n*_, which indicates the structural arrangement of *n* perovskite layers ABX_3_ that are enclosed by rock-salt-like layers AX (see Figure 1). Here, A is often a lanthanide or alkaline earth ion, and B, in most cases, is a transition metal cation. X is the anion, generally oxygen. In the past years, mixed anionic materials like oxyfluorides, -nitrides, or -hydrides have gained increased interest.^1−3^

Topochemical fluorination of perovskite-type metal oxides allows the synthesis of new oxyfluorides with strongly modified physical properties like superconductivity^4^ or photocatalytic activity.^5^ Fluorination can be performed in different ways, and it can be classified in terms of the reaction temperature. High-temperature fluorinations, e.g., by reacting binary fluorides and oxides, are rarely used because of the high stability of many fluorides like strontium- or lanthanum (oxy)fluoride.^6,7^ Among the few examples of compounds obtained via a classic solid-state reaction is ANbO_2_F (A = Na, K).^8^ Additionally, high-pressure-assisted fluorination was performed to synthesize KTiO_2_F and PbMO_2_F (M = Sc, Mn).^9−11^ In contrast, low-temperature fluorination has a much higher potential, e.g., enabling the synthesis of metastable oxyfluorides. Common fluorination agents are fluorine gas,^12,13^ XeF_2_,^14^ NH_4_F,^15,16^ AF_2_ (A = Ni, Cu, Zn, Ag),^17,18^ or highly fluorinated polymers like poly(vinylidene fluoride) (PVDF)^19^ or polytetrafluoroethylene (PTFE).^20^ Especially, the use of fluorinated polymers has become popular as they are easy to handle and possess low toxicity, and the resulting oxyfluorides are free of inorganic residues as only volatile byproducts (CO_2_, CO, H_2_O, etc.) are formed during the reaction.

The focus of RP oxyfluoride research has been on the synthesis of new *n* = 1 compounds ever since, in 1994, the fluorination of Sr_2_CuO_3_ was found to induce superconductivity in Sr_2_CuO_2_F_2+δ_.^21^ In contrast, the number of known *n* = 2 RP oxyfluorides is very limited (only 12 entries in the ICSD database, release 2024.2^22^). Stable *n* = 2 oxyfluorides are, for example, Sr_3_Ti_2_O_5_F_4_^23^ and Ln_1.2_Sr_1.8_Mn_2_O_7_F_2_ (Ln = Pr, Nd, Sm, Eu, and Gd)^24^ (obtained from the reaction of corresponding oxides with PVDF), as well as La_2_SrCr_2_O_7_F_2_^17^ and Sr_3_(M_0.5_Ru_0.5_)_2_O_7_F_2_ (M = Ti, Mn, Fe)^25^ (obtained from fluorination with CuF_2_). One reason for the small number of *n* = 2 oxyfluorides is the difficulty in isolating phase-pure starting oxides. With increasing n, the energetic differences between the various phases decrease, and therefore, mixtures are often formed. In many cases, the formation of the *n* = 1 and *n* = ∞ (perovskite) members is preferred. In the K_2_NiF_4_-structure type (*n* = 1) and the three-dimensional perovskite (*n* = ∞), only one crystallographic A-site exists. In the higher members of the RP series, there are (at least) two distinct cationic sites (12-fold coordinated in the perovskite blocks and 9-fold coordinated at the interface of the (AO) and (ABO_3_) layers, respectively; see Figure 1). One way of stabilizing *n* > 1 RP phases therefore is the incorporation of different A-cations with deviating sizes. If heterovalent A-type ions are used (e.g., substitution of Ln^3+^ by Sr^2+^), the oxidation state of the B cation changes, or oxygen vacancies form. Both effects can help to stabilize higher RP phases, too, because B cations with different oxidation states possess deviating radii, and oxygen vacancies will show site preferences as well. Again, for the *n* = 1 and the *n* = ∞ members, only one crystallographic B position exists, while in the *n* > 1 phases, at least two sites can be distinguished. Analogous reasoning can be applied to the X-sites as well (please note that these considerations refer to the archetype structures of the highest symmetry and ignore structural distortions).

In the case of *n* = 3 oxyfluorides, similar difficulties occur in the preparation of the starting oxide. While there are several *n* = 3 lanthanide nickel oxides like Pr_4_Ni_3_O_10_,^26^ Nd_4_Ni_3_O_10_,^27^ etc., described in literature, La_4_Ni_3_O_8_F and La_4_Ni_3_O_8_F_2_ are the only known *n* = 3 oxyfluorides to date. These compounds were prepared by solvothermal fluorination of La_4_Ni_3_O_8_ with XeF_2_, but besides X-ray diffraction (XRD), no further characterization of the physical properties has been performed.^28^

Figure 1 shows the highest symmetric structure (space group *I4/mmm*) of an *n* = 3 RP oxide. Four different anionic sites can be distinguished, denoted as central apical (ca, Wyckoff position 4*e*, (*0,0,z*_*1*_)), terminal apical (ta, Wyckoff position 4*e*, (*0,0,z*_*2*_)), central equatorial (ce, Wyckoff position 4*c*, (*1/2,0,0*)), and terminal equatorial (te, Wyckoff position 8*g*, (*1/2,0,z*_*3*_)). Additionally, all RP oxides possess (usually unoccupied) interstitial anionic sites (i, Wyckoff position 8*f*, (*1/4,1/4,1/4*)) within the AX rock salt layer, which can be occupied by up to two additional anions per formula unit. For the oxyfluorides, F^–^ is reported to prefer the (terminal) apical sites.^5,29−33^ The second most preferred sites are the interstitial ones, but there are also reports about fluoride on both interstitial and apical sites.^1,17,29,34^ Fluoride ions on equatorial sites have not been reported yet, but there are few experimental works considering a possible statistical occupation on all anionic sites.^25,30^

In this work, we present the synthesis of the new *n* = 3 RP oxyfluoride La_0.5_Sr_3.5_Fe_3_O_7.5_F_2.6_ obtained from fluorination of La_0.5_Sr_3.5_Fe_3_O_10−δ_ with PVDF. La_0.5_Sr_3.5_Fe_3_O_7.5_F_2.6_ crystallizes in space group *Pbca*, and the structure was solved by joint Rietveld refinements of neutron and X-ray powder diffraction data in combination with the results of elemental analysis methods (XRF, F-ISE, and iodometric titration). The magnetic structure was characterized by refinement of the magnetic space group from neutron powder diffraction data. A G-type antiferromagnetic ordering was found, and an unusually high Néel temperature above room temperature was derived from temperature- and field-dependent magnetic measurements, as well as temperature-dependent Mößbauer spectroscopy. Finally, a comparatively high decomposition temperature >700 °C was derived from temperature-dependent XRD studies.

## Experimental Section

### Synthesis

The oxyfluoride La_0.5_Sr_3.5_Fe_3_O_7.5_F_2.6_ was synthesized via a two-step synthesis. First, the corresponding RP oxide La_0.5_Sr_3.5_Fe_3_O_10−δ_ was prepared by classic solid-state synthesis. Stoichiometric amounts of La_2_O_3_ (VEB Jenapharm-Apolda, purity >99.6%, dried at 950 °C for 3 h and stored in a desiccator), SrCO_3_ (Sigma-Aldrich, ≥98%), and Fe_2_O_3_ (VEB Laborchemie Apolda, purity ≥ 98%) were ground in a planetary ball mill in an agate grinding jar with equivalent masses of isopropanol and agate balls for 18 h. The dried mixture was loaded in alumina crucibles and heated in a muffle furnace in air at 1400 °C for 12 h (heating rate 7 K/min) and afterward cooled down to room temperature in the furnace. The product absorbs water from air (as described by Lehtimäki et al.^35^) and was therefore stored in a desiccator over sodium hydroxide.

The oxyfluoride was prepared by mixing La_0.5_Sr_3.5_Fe_3_O_10−δ_ with 150 mol % PVDF ((CH_2_CF_2_)_*n*_ according to the formula mass of the repeating unit (M(CH_2_CF_2_) = 64.03 g/mol)) in an agate mortar. The oxide molar mass has been assumed as non-oxygen-deficient (δ = 0), resulting in a slightly higher amount of PVDF, comparable with reported excesses.^23,34^ The oxide/PVDF mixture was then heated in a glazed crucible in air at 5 K/min to 480 °C and kept at this temperature for 12 h. The resulting product was homogenized and stored in a desiccator to prevent the reaction with moisture.

### Characterization

Room-temperature powder XRD measurements in the range 2θ = 10–120° were performed on a Bruker AXS D8 Advance diffractometer operating with Cu–K_α1,2_ radiation (λ = 1.542 Å) and a silicon strip detector (LynXEye). Temperature dependent measurements up to 950 °C were conducted on a STOE STADI MP diffractometer with monochromatic Mo–K_α1_ radiation (λ = 0.709 Å) equipped with a DECTRIS MYTHEN2 1K detector and a STOE capillary furnace.

Neutron diffraction (ND) data of the oxyfluoride (ca. 0.8 g) were recorded at 300 K on the high-resolution powder diffractometer D2B at the Institute Laue-Langevin in Grenoble, France. Two different wavelengths of λ = 1.594 Å and λ = 2.398 Å were used with measuring times of about 3.0 and 5.25 h, respectively.^36^

Joint Rietveld refinements of XRD and ND data were performed using GSAS-II software.^37^ Peak shape parameters were determined from the refinement of an α-Al_2_O_3_ reference scan. Magnetic reflections were determined with GSAS-II using the kSUBGROUPSMAG tool, which accesses the data of the Bilbao Crystallographic Server.^38^ Bond valence sum (BVS) calculations were carried out using the software BondStr (Version: July 2010).

Magnetic measurements were performed with the ACMS option of a Quantum Design PPMS-9. Approximately 100 mg of the samples (oxide and oxyfluoride) was filled in gelatin capsules, ensuring a low diamagnetic contribution to the obtained susceptibilities. The temperature-dependent moment was measured at an external field of 2 T in the temperature range of 5–300 K, applying zero-field-cooled (ZFC) and field-cooled (FC) conditions. Field dependence of the magnetic behavior was analyzed by recording the complete hysteresis from 5 to −5 T at both 5 and 300 K.

The oxygen content of the oxide was determined from thermogravimetric analysis (TGA) on a TA Instruments TGA550 thermobalance in flowing forming gas (oven gas 10% H_2_ in N_2_, 50 mL/min; balance protecting gas: N_2_ 50 mL/min). Samples of about 20 mg were heated to 1000 °C at 10 K/min and held for 2 h to ensure complete reduction of iron.

The cationic composition of the precursor oxide was quantified by X-ray fluorescence (XRF) spectroscopy using a Panalytical Epsilon 4 spectrometer. The analysis was performed using the fundamental parameter method (*Omnian* mode) based on 6 independent scans with excitation voltages between 5 and 50 kV in combination with filters of different materials and thicknesses (Ag, Cu, Al, and Ti).

IR spectra were recorded with a Bruker Tensor 27 instrument equipped with a diamond ATR unit.

The amount of fluoride was quantified using a Mettler Toledo SevenMulti ion-sensitive electrode (ISE). About 10 mg of the sample was dissolved in 5 M HCl in polymethylpentene volumetric flasks. Iron +III was reduced to +II with hydroxylamine hydrochloride solution (4 g/L) to avoid systematic errors by [FeF_6_]^3–^ formation.^39,40^ The dissolved cations were additionally complexed using Titriplex IV (2-fold excess per cation). The pH value was adjusted to pH ≈ 6 against bromothymol blue by the addition of sodium hydroxide solution and an acetic acid/sodium acetate buffer. The F-content was obtained for three independent solutions by a five-point standard addition method (5 times 1 mL of 100 μg F^–^/ml standard solution (Mettler Toledo)).

Average iron oxidation state of the oxyfluoride was determined by iodometric titration performed under flowing argon. About 20 mg of the sample, excess KI, and 1 g of Na_2_CO_3_ were dissolved in 10 mL of 5 M HCl. For titration, a 5 mM Na_2_S_2_O_3_ solution was used, and the oxidation state was averaged from three independent measurements per sample.

Room-temperature Mößbauer spectra were recorded in transmission geometry in constant acceleration mode on a WissEL (Wissenschaftliche Elektronik GmbH) spectrometer, equipped with a ^57^Co(Rh) source (Ritverc JSC) having a nominal activity of 50 mCi and a 10 mm active window (sealed by Be) that is kept at room temperature. The polycrystalline material was filled in a polyetheretherketone (PEEK) container, and PTFE-made disks were used to ensure homogeneous distribution of the sample within the containment. Incoming signals were detected with a proportional counter and cached in a multichannel analyzer (CMCA-550, operating in 512 channels) that transferred counts to the Wissoft 2003 interface.^41^ Isomer shifts are reported relative to α-Fe foil at 298 K (without correction in terms of the second-order Doppler shift). Suitable fit models could be obtained with the Recoil software package.^42^

## Results and Discussion

**Figure 2:**
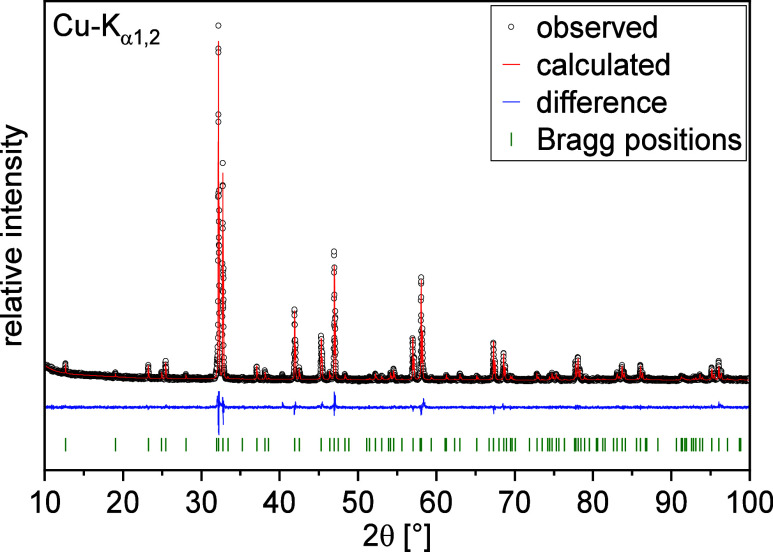
XRD Rietveld refinement of La_0.5_Sr_3.5_Fe_3_O_8.75_.

The precursor oxide La_0.5_Sr_3.5_Fe_3_O_10-δ_ was obtained as a grayish-black powder. Its cationic composition was determined via XRF and a molar ratio of La/Sr/Fe (0.5:3.6:3) was obtained, which is in good agreement with the nominal one. Therefore, in the structure refinements, the site occupation factors (SOFs) of the iron positions were fixed to unity. Furthermore, the A-sites were assumed to be fully occupied, and only the Sr/La ratio was allowed to vary. The SOFs were constrained to yield the known La/Sr content. Rietveld analysis (Figure 2) was performed in the highest symmetric space group *I4/mmm*. The detailed structural parameters can be found in Table 1. The obtained cell parameters are in agreement with those of similar *n* = 3 RP oxides.^43−47^ Lanthanum seems to slightly prefer the cationic sites within the rock salt layer (15%) compared to the perovskite layer (10%). In contrast, Lee et al. reported a statistic distribution.^44^ Similar statistic distributions were reported for *n* = 2 analogues.^48−50^ Although the enrichment of La on the La2/Sr2 site is small (12.5% corresponds to a statistical arrangement), we believe that our result is reliable because the atomic scattering factors of Sr^2+^ and La^3+^ differ by about 50%.^51^ Thus, the difference is five times its standard deviation. Compared to LaSr_3_Fe_3_O_10−δ_ with 0.1 ≤ δ ≤ 0.8,^44^ the here presented oxide (La_0.5_Sr_3.5_Fe_3_O_10−δ_) is expected to have a lower oxygen content. Based on our refinement, all oxygen sites show an occupation between 83 and 100%, resulting in the formula La_0.5_Sr_3.5_Fe_3_O_8.75_, which corresponds to iron in the oxidation state (+III).

**Table 1:** Structural Parameters of La_0.5_Sr_3.5_Fe_3_O_8.75_ Obtained by Rietveld Refinement of the X-ray (Cu–K_α1,2_) Powder Diffraction Data^a^

atom	Wyckoff position	*x*/*a*	*y*/*b*	*z*/*c*	SOF	*U*_iso_(Å^2^)
Fe1	2*a*	0	0	0	1	0.0181(17)
Fe2	4*e*	0	0	0.1404(13)	1	0.0207(10)
Sr1/La1	4*e*	0	0	0.5703(7)	0.903/0.097(10)	0.0176(9)
Sr2/La2	4*e*	0	0	0.7013(6)	0.847/0.153(10)	0.0222(10)
O1 (te)	8*g*	0	0.5	0.1364(27)	0.834(7)	0.0200
O2 (ca)	4*e*	0	0	0.0718(5)	0.858(14)	0.0200
O3 (ta)	4*e*	0	0	0.2091(31)	0.979(15)	0.0200
O4 (ce)	4*c*	0	0.5	0	0.871(15)	0.0200

a Space group *I4/mmm*; *a* = 3.8680(12) Å; *c* = 28.0168(15) Å; cell volume = 419.2(3) Å^3^. *R*_w_ = 3.82%, χ^2^ = 2.13, GOF = 1.46.

**Figure 3:**
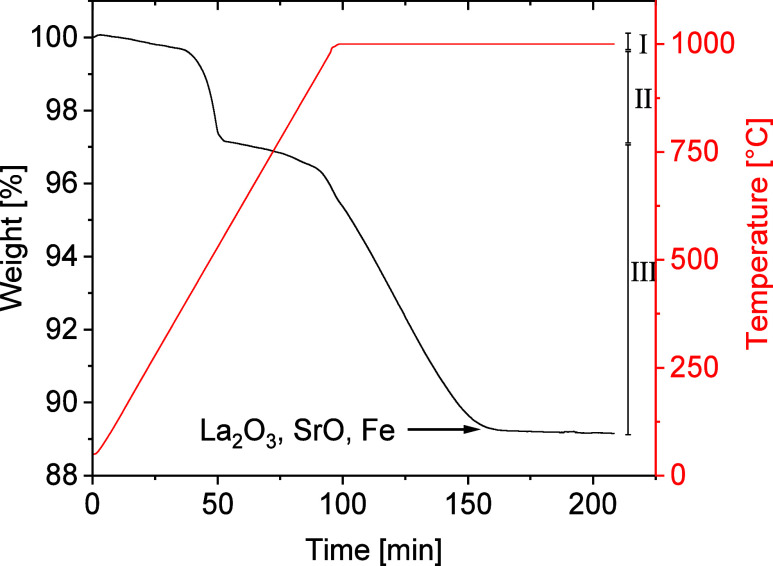
TGA of the reduction of La_0.5_Sr_3.5_Fe_3_O_8.75_ in forming gas. The three reduction steps are marked.

Thermogravimetric measurements in a reducing atmosphere were performed to validate the oxide oxygen content. Neither lanthanum(+III) nor strontium(+II) is reduced by forming gas at 1000 °C, while iron(+III) is reduced to its metallic state.^52^ Consequently, after reduction, a mixture of La_2_O_3_, SrO, and Fe was identified by X-ray diffraction. As shown in Figure 3, the reduction proceeds in three steps. The first small step (weight loss of 0.39% in the temperature range 50–400 °C) is most likely due to the release of chemisorbed species like OH^–^ or CO_3_^2–^ (as H_2_O and CO_2_, respectively).^35^ The second step (Δ*m* = 2.42%, *T*_onset_ = 453 °C) is roughly in accordance with the reduction of Fe(III) to Fe(II) (expected Δ*m* = 3.5%). The last step has an onset temperature of about 942 °C and was not finished when the maximum temperature of 1000 °C was reached. Instead, prolonged heating for more than 1 h was required to obtain the final total weight loss of 10.76%. This value is in very good agreement with the expected one for the reaction of La_0.5_Sr_3.5_Fe_3_O_8.75_ to SrO, La_2_O_3_, and Fe (4.5 O per f.u. corresponding to 10.53%) even if one subtracts the first weight loss (0.39%). Thus, our TGA results confirm the oxygen content of 8.75 and the oxidation state of + III for iron.

**Figure 4:**
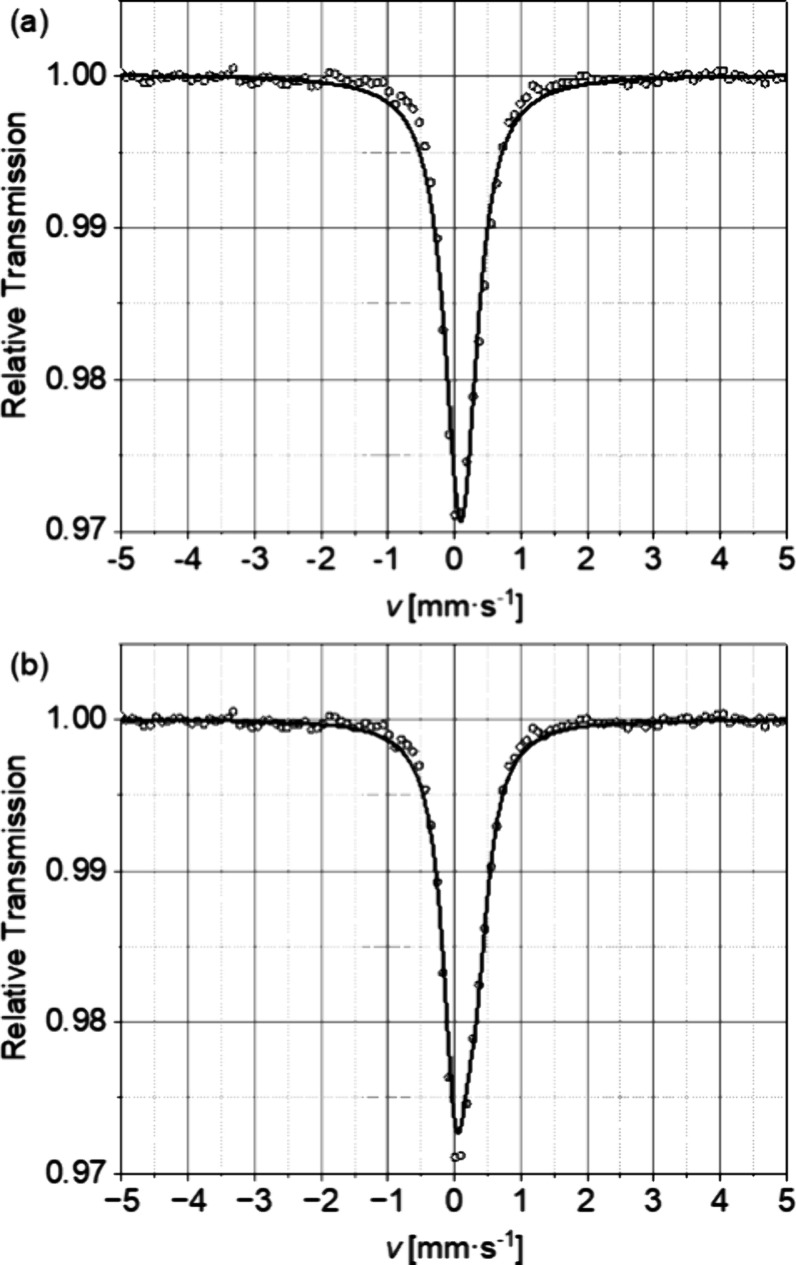
Room-temperature ^57^Fe Mößbauer spectra of La_0.5_Sr_3.5_Fe_3_O_8.75_. (a) Broadened singlet and (b) asymmetric doublet.

The iron oxidation state of the oxide was further investigated by ^57^Fe Mößbauer spectroscopy, as shown in Figure 4a. The obtained room-temperature spectrum can be fitted by one broadened singlet with a small positive isomeric shift δ = 0.098(4) mm s^–1^ in line with high-spin iron(III) in an octahedral environment. The large width of Γ = 0.558(12) mm s^–1^ may indicate relaxational broadening or multisite character. An alternative simulation in terms of a nonsymmetric doublet, characteristic of relaxation spectra, gives likewise good fits (see Figure 4b). As a matter of fact, the presence of oxygen vacancies necessarily renders the environment of the ferric iron centers anisotropic, yielding the O_6−δ_ coordination with the statistic orientation of the resulting (local) square pyramids. Even with the oxygen vacancies, the environment cannot be very anisotropic, as the quadrupole splitting is quite small.

**Figure 5:**
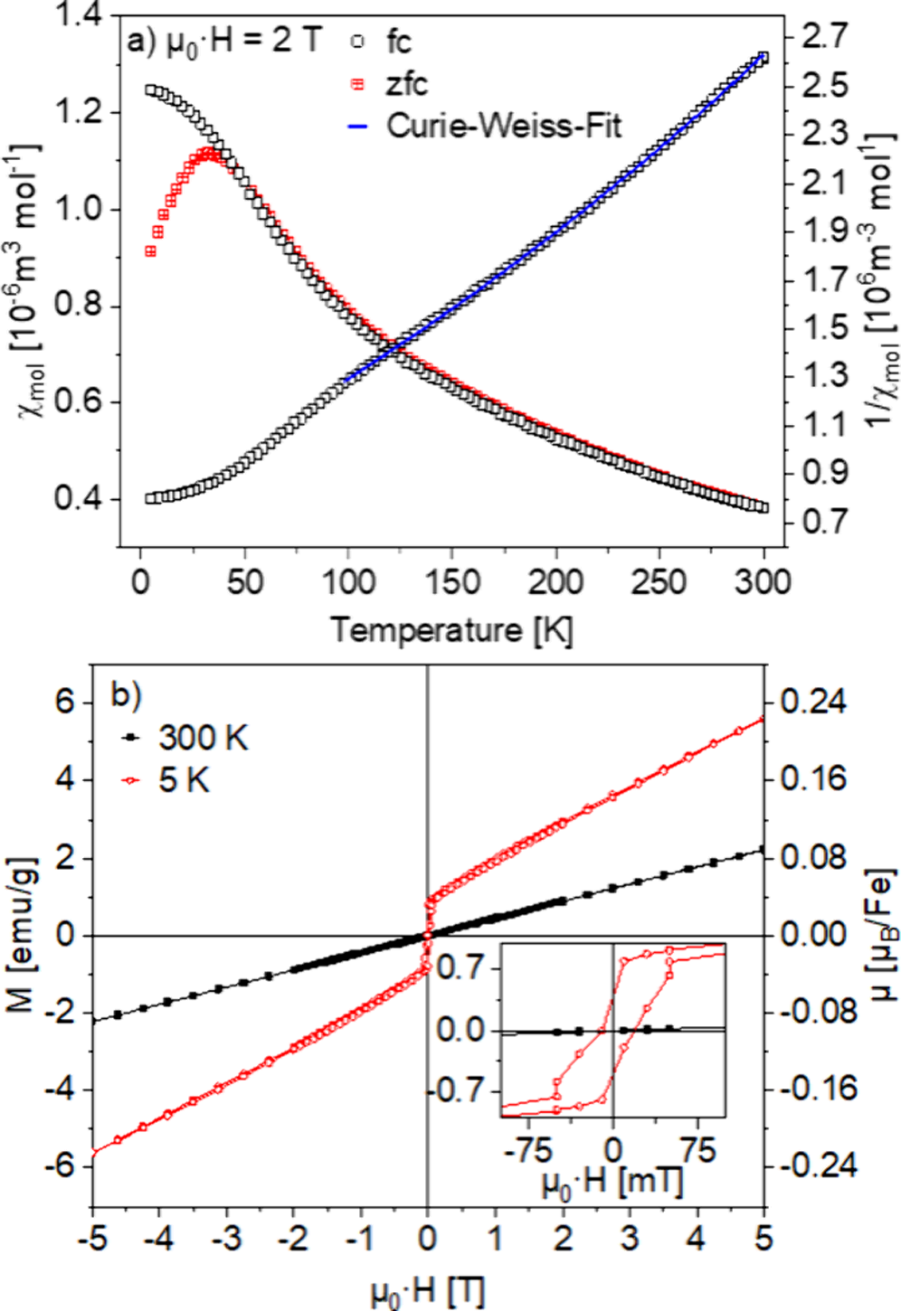
(a) Temperature-dependent susceptibility of La_0.5_Sr_3.5_Fe_3_O_8.75_ in an external field of 2 T. (b) Field-dependent magnetic moment at 300 and 5 K. The inset shows a detailed view of the low-field region.

Figure 5a shows the temperature dependence of the magnetic susceptibility measured in an external field of 2 T. A splitting of the FC/ZFC data below about 50 K possibly may result from a frustrated spin arrangement. This correlates with a small hysteresis found at 5 K in the field-dependent measurement at FC conditions, as shown in Figure 5b.

The inverse susceptibility between room temperature and 50 K was fitted with an extended Curie–Weiss law (eq 1). From the obtained Curie constant of 212 cm^3^ K mol^–1^, a magnetic moment of 6.7 μ_B_ is obtained. This value is surprisingly much larger than the 5.92 μ_B_ expected for Fe^3+^ in high-spin configuration. From Mößbauer spectroscopy, the presence of iron(III) in an isotropic environment (on average) was deduced, and no magnetic multiplet structure was observed. For the ^6^S ground state of high-spin (hs) Fe^3+^, no effect of the crystal field is expected, either. It has to be noted, however, that the Curie–Weiss law only applies to isolated ions. The observed enhanced effective moment might therefore emanate from magnetic interactions between the iron centers. Furthermore, the value of *C* and, in turn, of μ_eff_ strongly depend on the temperature interval used for the fit, resulting in a rather large uncertainty of the results. The large negative Weiss temperature of θ ≈ −137 K indicates a strong antiferromagnetic interaction of the magnetic moments.
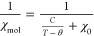
1

The magnetic field dependence of the magnetization is shown in Figure 5b. At room temperature, linear behavior is found, typical for a paramagnet. This is in accordance with the singlet in the room-temperature Mößbauer spectrum. At 5 K, a narrow magnetic hysteresis occurs with a coercivity of ±75 mT. Apart from this, a linear behavior dominates the magnetization data. A small saturation magnetization of 1.11 emu/g (0.13 μ_B_/f.u., respectively 0.045 μ_B_/Fe)) can be estimated from an extrapolation toward μ_0_·*H* = 0 T of the linear fit of the high-field region (μ_0_·*H* ≥ 3.5 T). A similar behavior has already been reported by Li for LaSr_3_Fe_3_O_8.68_ both at RT and at 5 K.^53^ It was interpreted as a weak ferromagnetic component. We emphasize that such effects can in principle also arise from minor impurities in the sample. In our material, this possibility can most likely be excluded as different batches show identical behavior. The small hysteresis at 5 K, together with the strongly negative Weiss constant, can be interpreted as a weak ferrimagnetic moment, resulting from a canted antiferromagnetic spin arrangement.^54^

### Formation and Characterization of the Oxyfluoride

**Figure 6:**
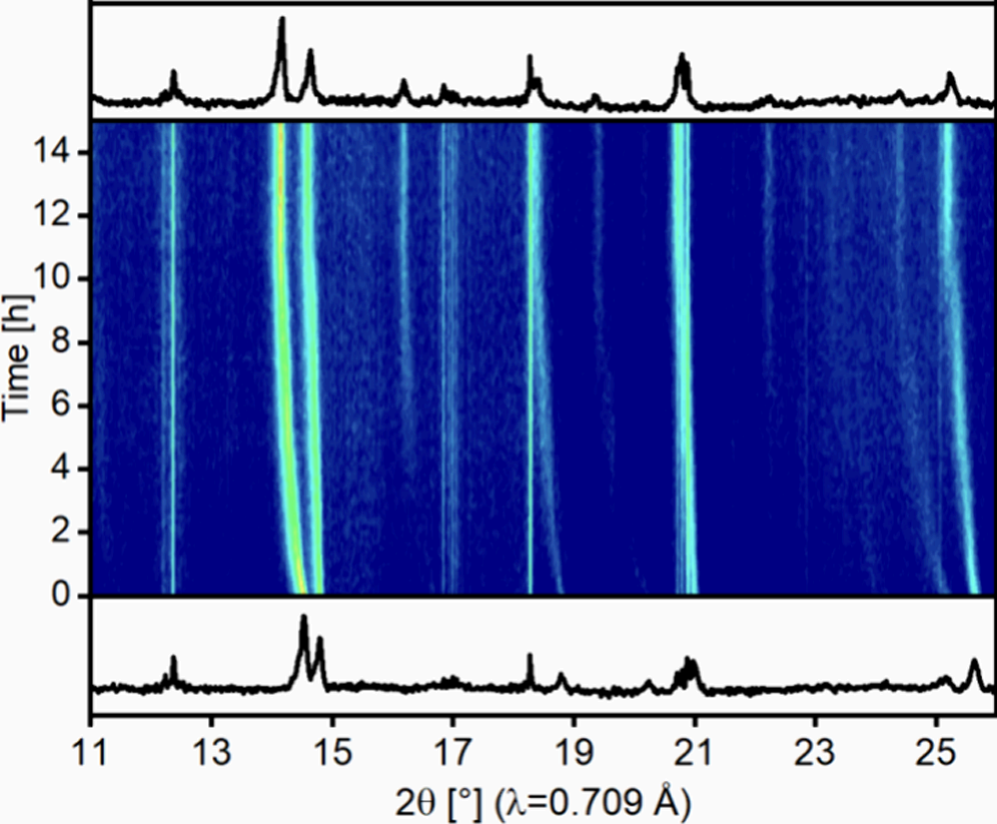
Time-dependent in situ XRD of the topochemical fluorination process at 340 °C. Bottom and top diffractograms show the initial and final patterns. Sharp and time constant peaks stem from the furnace.

Temperature-dependent XRD allows gaining information on the fluorination process, as shown before for La_2_NiO_2.5_F_3_ and La_2_CuO_3_F_2_.^1,54^Figure 6 shows the reaction of the precursor oxide with PVDF at 340 °C. This temperature was chosen because, at 480 °C (see the Experimental Section), the reaction time is rather short, and long XRD scans (15 min) had to be applied due to the strong Sr fluorescence background. At the beginning of the fluorination, a shift of several reflections is observed. Especially, the oxide (017)-reflex (2θ = 14.6°) is shifted toward smaller angles, indicating a strong elongation of the crystallographic *c*-axis. This can be explained by an insertion of fluoride ions on interstitial or apical positions. While the former possibility leads to a widening of the rock salt type layer, the latter results in an elongation of the MX_6_ octahedra. After approximately 10 h, new reflections of the final oxyfluoride appear, for example, at ca. 16.2° (119), 19.4° (0014), and 22.2° (0016). At the chosen temperature, the reaction is completed after approximately 12 h. The absence of reaction intermediates is highly interesting as, for La_2_NiO_2.5_F_3_ and La_2_CuO_3_F_2_, a whole number of less fluorinated intermediates are found.^1,54^

**Figure 7:**
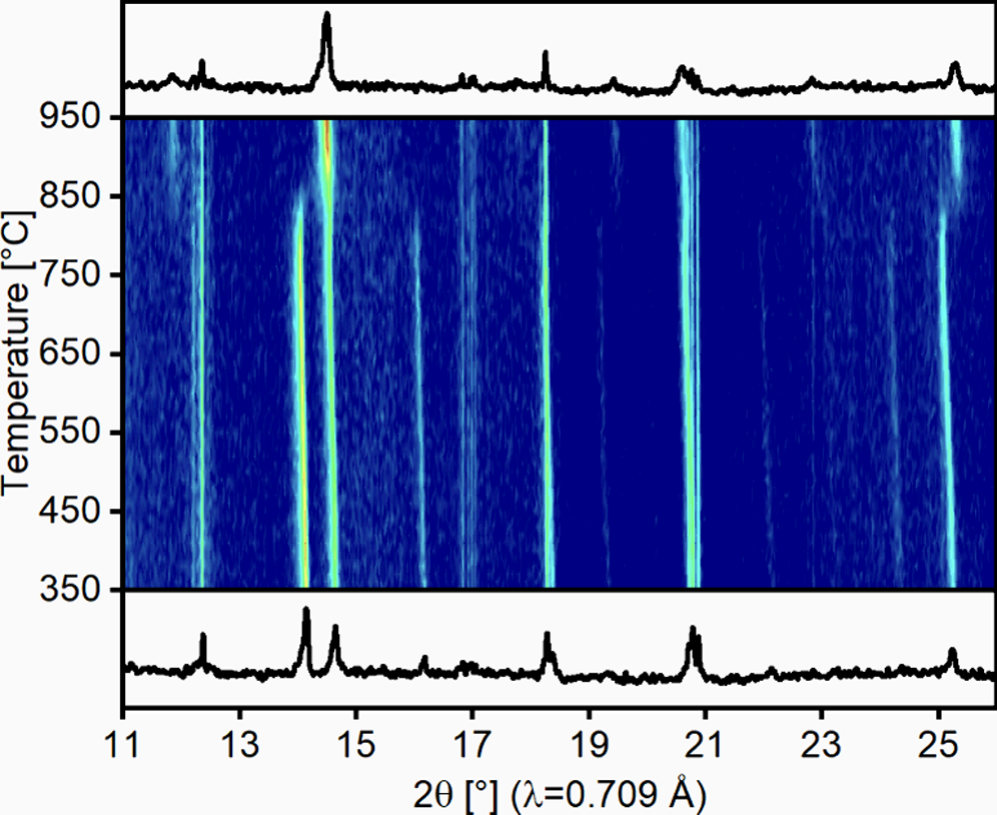
Temperature-dependent in situ XRD of the decomposition process of La_0.5_Sr_3.5_Fe_3_O_7.5_F_2.6_ (heating rate 25 K/min). Bottom and top diffractograms show the initial and final patterns.

High-temperature XRD was also used to study the thermal stability of the oxyfluoride. Figure 7 shows the heating between 350 and 950 °C. Up to 700 °C, no change is observed besides the thermal expansion of the unit cell. Figure S1 shows the temperature-dependent change of the cell parameters. The *c*-parameter shows the largest absolute expansion with a nearly linear behavior. Also, the *a*-parameter increases almost linearly in the entire temperature range, while the value of *b* seems to converge above ca. 650 °C (as discussed below, the oxyfluoride crystallizes in the orthorhombic system). No indications for a possible phase transition were found. The thermal decomposition of the oxyfluoride starts at about 800 °C and is finished at ca. 900 °C. The resulting product consists of a mixture of the cubic perovskite phase (Sr,La)FeO_3_ and lanthanum oxyfluoride. The observed decomposition temperature is surprisingly high compared to values found for other RP oxyfluorides, which are usually in the range of 400–500 °C.^33,54^

The oxyfluoride was obtained as a black powder after fluorination. IR spectroscopy showed no residues of the fluorination agent (see Figure S2). Iodometric titration gave an average iron oxidation state of 3.07(7). The oxidation state of Fe is therefore preserved during fluorination, underlining the nonoxidative and nonreductive character of the reaction with PVDF.^55^ The fluoride content was determined using an F-ion-selective electrode by the standard addition method to be 6.73(9)%. This corresponds to 2.6 fluoride ions per formula unit. This value is smaller than the amount provided by PVDF (150 mol % corresponding to 3 F f.u.^–1^). Obviously, not all fluoride is incorporated, most likely because of the high reaction temperature of 480 °C.

**Figure 8:**
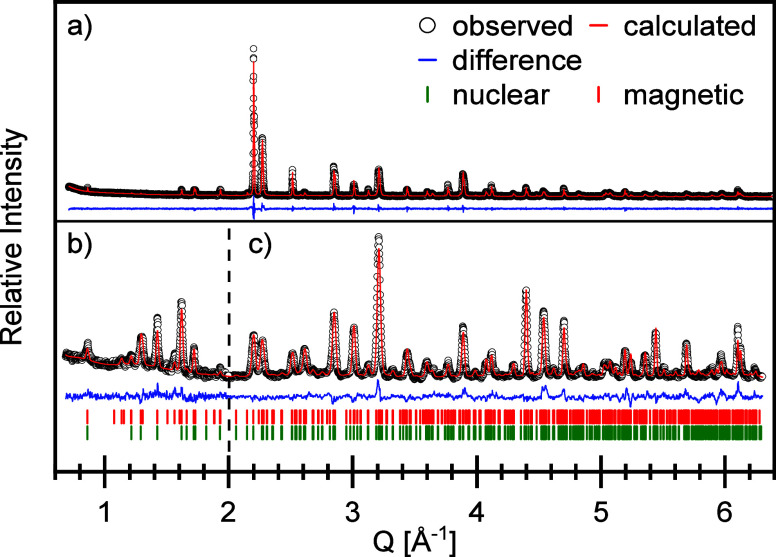
Joint Rietveld refinements of (a) X-ray (λ = 1.542 Å) and (b,c) neutron diffraction data (b: λ = 2.398 Å, intensity multiplied by a factor 3; c: λ = 1.594 Å). Green lines represent nuclear Bragg reflections, while red lines are associated with magnetic Bragg reflections (both SG: *Pbca*).

The crystal structure was solved by combined Rietveld refinement of X-ray and neutron powder diffraction data. Most of the reflections in the XRD pattern could be indexed by using a tetragonal unit cell (*I4/mmm*) with a significantly longer *c*-axis. On the other hand, a broadening of the (110) reflection points to an orthorhombic distortion. Refinements were therefore first conducted with an orthorhombic structure model in the space group *Fmmm*, which is the highest symmetric orthorhombic subgroup of *I4/mmm*. In the ND data recorded with λ = 1.594 Å, additional reflections at low *Q*-values, which could not be refined in this space group, were attributed to a magnetic contribution (see the magnetic structure). The region up to *Q* = 2 Å was therefore first omitted in the atomic structure refinement. In the resulting difference Fourier maps, missing electron densities at two sides of the central equatorial anion positions were seen as hints to a twisting of the Fe-X_6_ octahedra along the *c*-axis, which demands a less symmetric primitive space group. The final structural refinement was therefore performed in the orthorhombic space group *Pbca* (SG 61). The corresponding Rietveld plot is shown in Figure 8, and the structural data are given in Table 2. For the sites Sr1/La1 and Sr2/La2, identical *U*_iso_ values were used to reduce the number of refined parameters. For the anionic sites, the same strategy was pursued. The total amount of fluoride was restricted to the known value based on the ISE measurements. The SOFs of Sr and La were not refined, instead the values from the oxide precursor were taken. The occupation of the anionic sites was refined, and partially unoccupied terminal equatorial (*X*_te_) and central apical (*X*_ca_) sites were found, which is to be expected, as these sites are also partially unoccupied in the precursor oxide. The SOF values of F1 and O4 were found not to significantly deviate from full occupation. These values were therefore fixed to unity in order to minimize the number of refinable parameters. Additional electron density was found to be located on the interstitial anionic sites. Refinement of the SOFs gave an occupation of ∼30% of the interstitial site, yielding the sum formula La_0.5_Sr_3.5_Fe_3_(OF)_10.1(3)_. Based on this information, the ISE values and the oxidation state of (III) for iron (resulting from the iodometric titration) and the formula La_0.5_Sr_3.5_Fe_3_O_7.5_F_2.6_ can be deduced.

**Table 2:** Structural Parameters for La_0.5_Sr_3.5_Fe_3_O_7.5_F_2.6_ Obtained by Joint Rietveld Refinement of X-ray (λ = 1.542 Å) and Neutron (λ = 1.594 and 2.398 Å) Powder Diffraction Data^a^

atom	Wyckoff position	*x*/*a*	*y*/*b*	*z*/*c*	SOF	*U*_iso_(Å^2^)
Fe1	4*b*	0	0	0.5	1	0.031(1)
Fe2	8*c*	0.4944(19)	0.0064(15)	0.13172(8)	1	0.022(9)
Sr1/La1	8*c*	0.0000(12)	–0.0018(15)	0.0694(5)	0.903/0.097	0.021(5)
Sr2/La2	8*c*	0.0134(16)	0.0110(14)	0.1970(4)	0.847/0.153	0.021(5)
O1 (te)	8*c*	0.2560(4)	0.2720(3)	0.1407(6)	0.869(24)	0.024(5)
O2 (te)	8*c*	0.2560(4)	0.2409(31)	0.3612(5)	0.932(22)	0.024(5)
O3 (ca)	8*c*	0.4700(3)	0.0183(29)	0.0666(22)	0.927(8)	0.024(5)
O4(ce)	8*c*	0.2186(27)	0.3027(26)	0.4934(4)	1	0.024(5)
F1 (ta)	8*c*	0.0160(5)	0.0710(25)	0.2840(26)	1	0.040(3)
F2 (i)	8*c*	0.2180(16)	0.2650(10)	0.2518(16)	0.294	0.040(3)

a Space Group: *Pbca*. Cell parameters: *a*: 5.53740(8) Å, *b*: 5.54407(8) Å, *c*: 29.25407(18) Å, cell volume = 898.09(8) Å^3^. *R*_w_ = 3.77%, χ^2^ = 1.48, GOF = 1.22.

As oxygen and fluorine cannot be distinguished by X-ray or neutron diffraction, the anion distribution was investigated by BVS calculations. BVS values for different possible anionic orderings have been tested, and the results are listed in Table 3. The most reasonable BVS values were obtained with fluorine occupying the terminal apical (ta) octahedral positions and the additional interstitial anionic sites. This anion ordering scenario is similar to the ones found in the literature for analogue La/Sr/Fe-containing *n* = 1 and *n* = 2 RP oxyfluorides.^1,33^ For these compounds, it was found that fluorination always takes place as substitution on the terminal apical octahedral positions, which, as a result, yields strongly increased Fe-X_ap_ distances of about 2.5 Å, rendering the Fe-coordination environment pseudotetragonal pyramidal. Detailed atom distance information can be found in Table S1, while the coordination of the two iron centers is shown in detail in Figure S3, in comparison toward the parental oxide. The elongation of the bond length to ≈2.5 Å and the O3–Fe2–F1 angle of ≈170° are in good agreement with the *n* = 2 compound Sr_3_Fe_2_O_5.44_F_1.56_. In both compounds, the central atom of the FeO_5_F octahedra is shifted toward the apical oxygen ion.^6^ In contrast, the isostructural *n* = 1 oxyfluoride Sr_2_FeO_3_F has a significantly shorter Fe-X_ap_ distance of about 2 Å and the ideal X_ap_–Fe–X_ap_ angle of 180°, as well as very similar Fe–X distances of about 1.94 Å and a statistical distribution of O and F on the apical anionic position.^56^

**Table 3:** Results of BVS Calculations for Fully Fluorinated Anionic Sites (see Figure 1) with Given Global Instability Index (GII)^a^

atom site	bond valence sum for F on ... site						
	ta	ce	te2	te1	ca	statistical	
Fe1	3.25	2.83	3.25	3.25	3.04	3.06	
Fe2	2.63	2.77	2.58	2.57	2.65	2.58	
La1	2.24	1.97	2.17	2.18	2.06	2.07	
Sr1	1.94	1.72	1.87	1.88	1.78	1.79	
La2	2.39	2.73	2.60	2.55	2.73	2.64	
Sr2	2.10	2.38	2.27	2.24	2.38	2.29	
te1	2.15	2.15	2.15	**1.69**	2.15	2.15	**1.69**
te2	1.88	1.88	**1.48**	1.88	1.88	1.88	**1.48**
ca1	1.99	1.99	1.99	1.99	**1.57**	1.99	1.57
ce1	1.84	**1.41**	1.84	1.84	1.84	1.84	**1.41**
ta1	**0.73**	1.15	1.15	1.15	1.15	1.15	**0.73**
i1	**1.14**	**1.14**	**1.14**	**1.14**	**1.14**	1.50	**1.14**
GII [%]	9.53	16.87	17.24	18.74	19.03	18.66	

a Fluoride occupation of interstitial sites has not been changed for these calculations except statistical calculation. Fluorinated sites are shown in **bold**.

**Figure 9:**
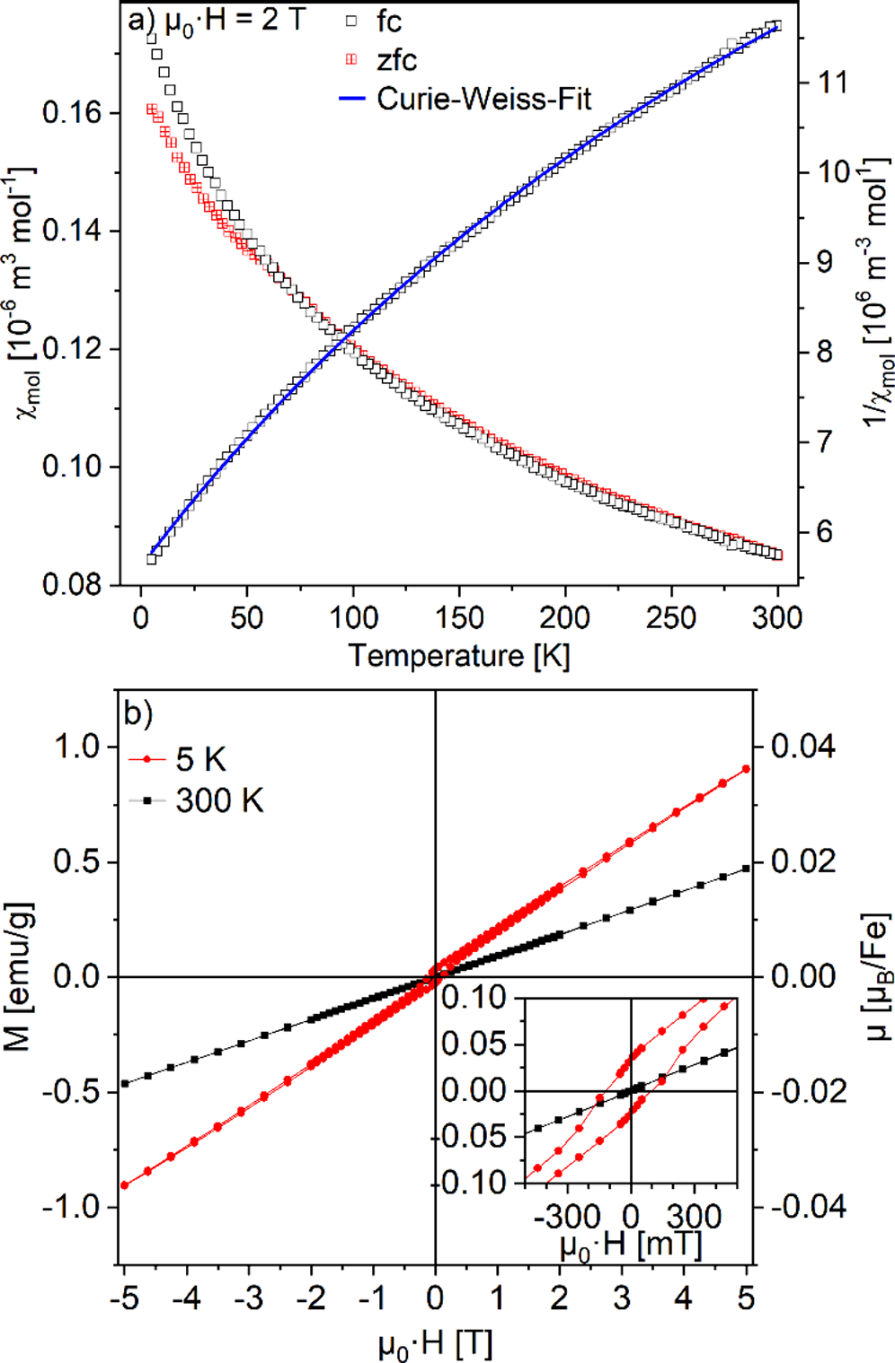
(a) Temperature-dependent susceptibility in an external field of 2 T. (b) Field-dependent magnetic moment of La_0.5_Sr_3.5_Fe_3_O_7.5_F_2.6_. The inset shows a detailed view of the low-field region.

The magnetic behavior of La_0.5_Sr_3.5_Fe_3_O_7.5_F_2.6_ was characterized by temperature- and field-dependent susceptibility measurements. As shown in Figure 9a, above ca. 60 K, ZFC and FC behavior are identical. Below this temperature, a weak splitting is observed. For the FC data, an extended Curie–Weiss fit (eq 1) was possible in the complete temperature region and led to a phenomenologically good description of the measured data. The Weiss temperature of −138(2) K is very similar to the value found for the oxide. A χ_0_ value of 0.0444(4) cm^3^ mol^–1^ points to a significant temperature-independent paramagnetic contribution. From the Curie constant of 18.0(2) cm^3^ K mol^–1^, a magnetic moment of about 1.95 μ_B_ per iron ion is obtained, indicating that only a small fraction of the iron centers follows a Curie–Weiss behavior. This might confirm the interpretation that the majority of spins are magnetically ordered. We would like to point out that the physical meaning of a Curie–Weiss fit below the magnetic ordering temperature (see below) should generally be interpreted with caution and requires complementary investigations. The fit is nevertheless interesting as it clearly shows deviations from the expected values and in particular from the results obtained for the corresponding oxide.

The field-dependent measurements depicted in Figure 9b show a basically linear dependence both at 300 and 5 K, which is in accordance with the antiferromagnetic ordering described below. At 5 K, an additional narrow hysteresis with a weak saturation moment of 0.045 emu g^–1^ (corresponding to 1.92 × 10^–3^ μ_B_/Fe) and a coercivity of 0.1 T was found. This low-temperature hysteresis may be caused by a minor ferrimagnetic component, resulting from spin canting.

**Figure 10:**
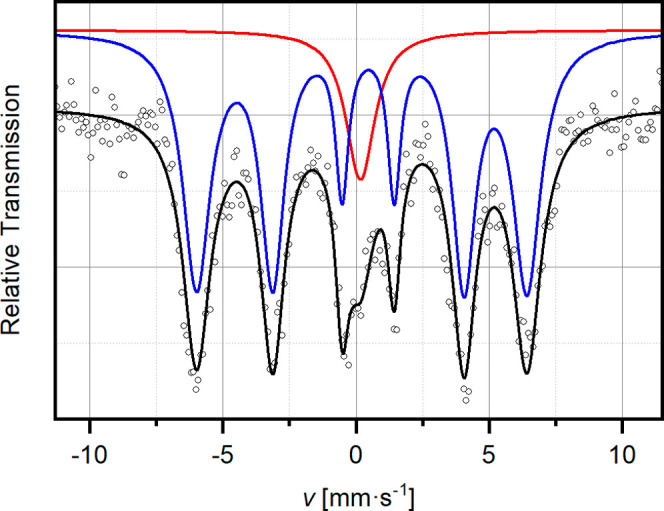
^57^Fe Mößbauer spectrum La_0.5_Sr_3.5_Fe_3_O_7.5_F_2.6_ at 298 K (open dots: measured data; black line: composite fit; red and blue lines: subspectra (shifted vertically for the sake of visual clarity)).

For further characterization of the magnetic properties of La_0.5_Sr_3.5_Fe_3_O_7.5_F_2.6_, a Mößbauer spectrum was recorded at 298 K. As can be seen in Figure 10, the spectrum is similar to the one reported by Tsujimoto et al. for the *n* = 2 RP oxyfluoride Sr_3_Fe_2_O_5.44_F_1.56_, which shows G-type antiferromagnetism.^6^ These authors have not modeled their spectra with individual multiplets but used a weighted overlay of a distribution of predefined sextets. Through this, decent fits could be obtained, but the results are not readily comparable to ours. The spectrum of La_0.5_Sr_3.5_Fe_3_O_7.5_F_2.6_ can be fitted with two components, namely, one broad singlet resonance at δ ≈ 0.17(9) mm s^–1^ with a width of Γ = 0.65(2) mm s^–1^ and one sextet (both types of subspectra, singlet and sextet, originate from species with a spin sextet ground state, ^6^S). The magnetic hyperfine interaction can be fitted with Γ = 0.28(8) mm s^–1^ and an effective field of 38.5(2) T. A slightly higher isomeric shift of δ = 0.34(2) mm s^–1^ compared to that of the precursor oxide (δ = 0.098 mm s^–1^) can be assigned to the higher electronegativity of fluoride and the consequential disturbance through the no longer isotropic d-electron distribution of the iron centers. From the δ values close to zero, the oxidation state of iron is again confirmed to be +III, in accordance with the iodometric results. The sextet component clearly dominates the spectrum with a weight of 87.2%. This supports the results from susceptibility measurements, indicating that most of the iron centers are magnetically ordered. The peak with a singlet appearance (weight 12.8%) has most likely the same origin as the small paramagnetic contribution (*C* = 1.8 × 10^–5^ m^3^ K mol^–1^) found in the susceptibility data.

According to preliminary high-temperature Mößbauer measurements, we estimate a Néel temperature above 523 K for La_0.5_Sr_3.5_Fe_3_O_7.5_F_2.6_ (see Figure S4). According to studies on FeF_3_, there is a 1/3 power law between the Mößbauer hyperfine field and the magnetic transition temperature.^57^ More detailed investigations are planned for the future and will be published separately, as they would exceed the scope of this article. High magnetic transition temperatures have been reported for a few other Fe-containing RP oxyfluorides. Tsujimoto et al. found an ordering temperature of 390 K for Sr_3_Fe_2_O_5.44_F_1.56_, and Oka et al. reported a transition temperature of about 490 K for Pb_3_Fe_2_O_5_F_2_.^6,58^ Unfortunately, none of these authors comment on the reasons for the strong increase of the ordering temperature. For the title compound, at least some possible explanations can most likely be ruled out. The fluoride incorporation does not alter the oxidation state of iron, and the equatorial anionic positions are not occupied by fluoride ions. Therefore, differences between the superexchanges Fe^3+^–O–Fe^3+^ and Fe^3+^–O–Fe^2+^(or Fe^4+^), respectively, Fe^3+^–O–Fe^3+^ and Fe^3+^–F–Fe^3+^, seem very unlikely as an origin.^59,60^

**Figure 11:**
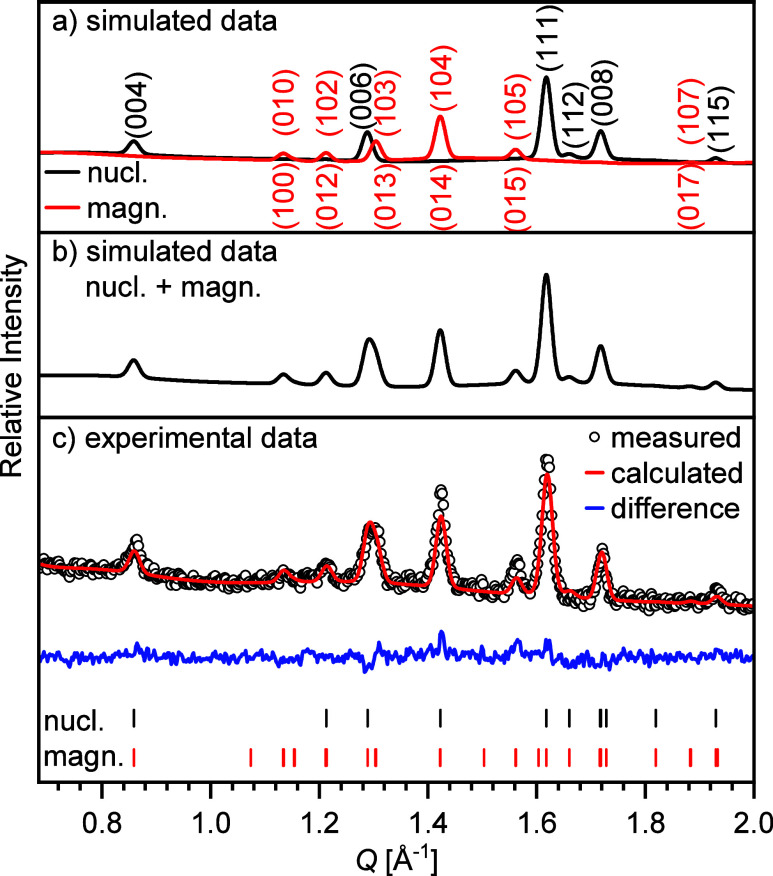
Comparison of simulated neutron diffraction data (λ = 2.398 Å) for (a) crystallographic and magnetic phase separately, (b) combination of both phases, and (c) experimental data.

**Figure 12:**
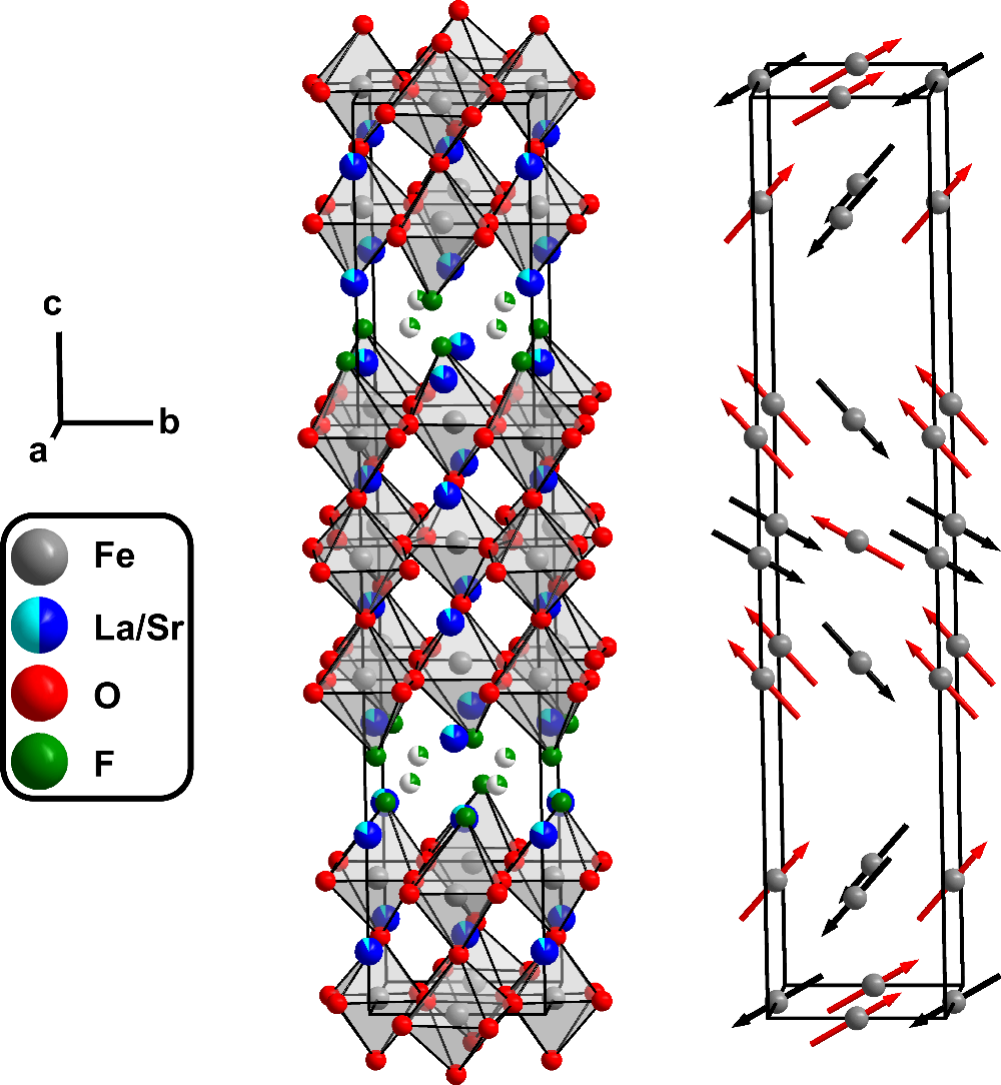
Crystal structure of La_0.5_Sr_3.5_Fe_3_O_7.5_F_2.6_ with representation of Fe-coordination polyhedra (left) and orientation of magnetic moments (right).

For the determination of the magnetic structure, additional ND data were collected with a wavelength of λ = 2.398 Å, giving a better signal-to-noise ratio at lower scattering vectors. All magnetic reflections can be indexed with the magnetic propagation vector *k* = 0. We therefore tried to refine the magnetic structure in all maximal magnetic subgroups of *Pbca* with magnetic moments at both Fe positions assuming Fe(III). The best result was obtained with the magnetic structure in the space group *Pbca* (SG#61.1.497 in Opechowski–Guccione notation^61^), which can be described as a type I magnetic subgroup (Fedorov group). The difference plot of this refinement is shown in Figure 11, together with the simulated intensities for the nuclear crystallographic phase and the magnetic phase (applying the same scale factor, instrument contribution, and background data). As a result of the magnetic refinement, a G-type antiferromagnetic ordering was deduced, and the results are presented in Table 4. The crystallographic and magnetic structures are shown in Figures 12 and S5. For both iron centers, the *x*-component of the magnetic vectors was found to be very small and did not converge to a stable value. Therefore, it was fixed to 0 in the final refinements, and only the *y* and *z* components were allowed to vary, leading to a stable refinement result. It has to be noted though that due to the limited number of magnetic reflections, the relative orientation of the *z*-component of both magnetic sites might be inverted as refinements with (Fe1 + *y* – *z*) (Fe2 + *y* + *z*) and (Fe1 + *y* + *z*) (Fe2 + *y* – *z*) gave almost identical results with the first case yielding slightly lower *R*-values (compare Figure S6 for alternative setting). The modulus of both centers lies between 1.92(6) μ_B_/Fe (Fe1, central octahedra) and 2.29(3) μ_B_/Fe (Fe2, terminal, F-containing octahedra). Thus, within experimental error, both moments are in accordance with the expected value of 2.5 μ_B_.

**Table 4:** Refined Magnetic Moments with Respect to the Crystallographic Axes^a^

atom	site symmetry	*M*_*x*_	*M*_*y*_	*M*_*z*_	modulus [μ_B_/Fe]	multiplicity
Fe1	–1	0	1.5(4)	–1.2(4)	1.92(6)	4
Fe2	1	0	1.8(2)	1.4(2)	2.29(3)	8

a *R*_w_ = 4.05%, χ^2^ = 1.66, GOF = 1.29.

In summary, from the ND and Mößbauer experiments, an antiferromagnetic ordering at room temperature is concluded. Based on preliminary high-temperature Mößbauer and EPR measurements, a value of 520 < *T*_N_ < 620 K is likely. The determination of the temperature-dependent magnetic structure will be the subject of further studies.

## Conclusions

The first iron-based *n* = 3 RP oxyfluoride La_0.5_Sr_3.5_Fe_3_O_7.5_F_2.6_ has been prepared by topochemical fluorination of the oxide La_0.5_Sr_3.5_Fe_3_O_8.75_. For both the precursor oxide and the resulting oxyfluoride, an oxidation state of +III for iron was found, highlighting a neither reductive nor oxidative character of the fluorination with PVDF at 480 °C in air. In addition, the oxyfluoride exhibits an unusually high thermal stability up to 800 °C. Joint Rietveld refinements of powder X-ray and neutron diffraction data were successful in the crystallographic space group *Pbca.* About 30% of the interstitial anionic position in the rock salt-type AX layer was found to be occupied by fluoride ions. Furthermore, the apical octahedral position of the terminal perovskite layer (i.e., the one neighboring the AX layer) is occupied by fluorine as derived from BVS calculations. Magnetic measurements show that in La_0.5_Sr_3.5_Fe_3_O_7.5_F_2.6_, only a small fraction of the iron spins follows a Curie–Weiss behavior, while the majority is magnetically ordered, as indicated by a magnetic hyperfine splitting observed in room-temperature Mößbauer spectroscopy. The magnetic structure was solved based on neutron powder diffraction data. A G-type antiferromagnetic ordering of the Fe(III) spin system with *T*_N_ > 450 K was found. The corresponding oxide shows a much lower ordering temperature, which can be identified below 50 K. Therefore, topochemical fluorination raises the magnetic ordering temperature by several hundred degrees and create a room-temperature stable antiferromagnetically ordered oxyfluoride by aliovalent substitution of oxygen by fluoride.

## Supporting Information Available

Temperature-dependent cell parameters based on the sequential Rietveld refinements of La_0.5_Sr_3.5_Fe_3_O_7.5_F_2.6_; FT-IR spectra of the precursor oxide with and without PVDF, pure PVDF, and the resulting oxyfluoride; atom distances for La_0.5_Sr_3.5_Fe_3_O_7.5_F_2.6_ based on room-temperature Rietveld refinements; coordination geometries of the Fe-X polyhedra for La_0.5_Sr_3.5_Fe_3_O_8.75_ and La_0.5_Sr_3.5_Fe_3_O_7.5_F_2.6_; and crystal structure of La_0.5_Sr_3.5_Fe_3_O_7.5_F_2.6_ with the representation of Fe-coordination polyhedra and orientation of the magnetic moments (PDF)
