# Supplementary material for: Structure and Magnetic Properties of the n = 3 Ruddlesden–Popper Oxyfluoride La0.5Sr3.5Fe3O7.5F2.6
Source: Inorg Chem. 2024 Oct 18;63(43):20427–37. doi: 10.1021/acs.inorgchem.4c02835 (PMC11523217; doi:10.1021/acs.inorgchem.4c02835)
Supplement: Supplementary file 1 — ic4c02835_si_001.pdf [file ic4c02835_si_001.pdf]

Structure and Magnetic Properties of the n = 3 Ruddlesden-Popper-Oxyfluoride $La_{0.5}Sr_{3.5}Fe_3O_{7.5}F_{2.6}$

Supporting Information

Andy Bivour[1], Jonas Jacobs[1], Florian Daumann[2,3], Gerald Hörner[2,3], Birgit Weber[2,3], Clemens Ritter[4], Stefan G. Ebbinghaus*[,1]

[1] Martin-Luther-University Halle-Wittenberg, Department of Chemistry, Inorganic Chemistry, Kurt-Mothes-Straße 2, D-06120 Halle, Germany

[2,3] Friedrich-Schiller-University Jena, IAAC, Humboldtstraße 8, 07743 Jena, Germany; experiments were performed at the Deparment of Chemistry, University of Bayreuth, D-95447 Bayreuth, Germany

[4] Institut Laue-Langevin, 71 Avenue des Martyrs, F-38042 Grenoble Cedex 9, France

* Corresponding Author

Email: stefan.ebbinghaus@chemie.uni-halle.de

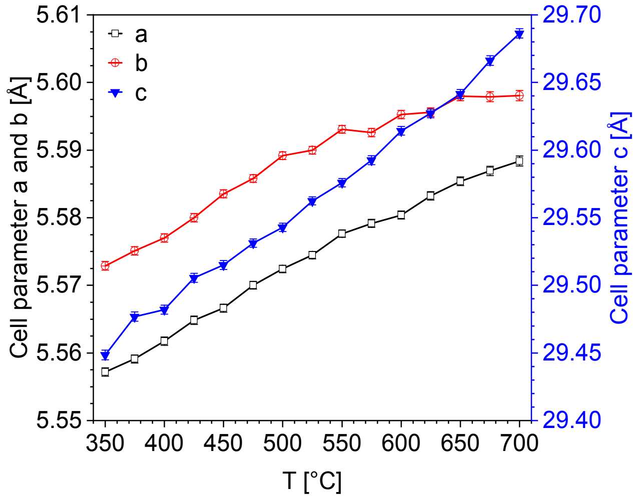

Figure S1: Temperature dependent cell parameters based on sequential Rietveld refinements between 350 and 700 °C.

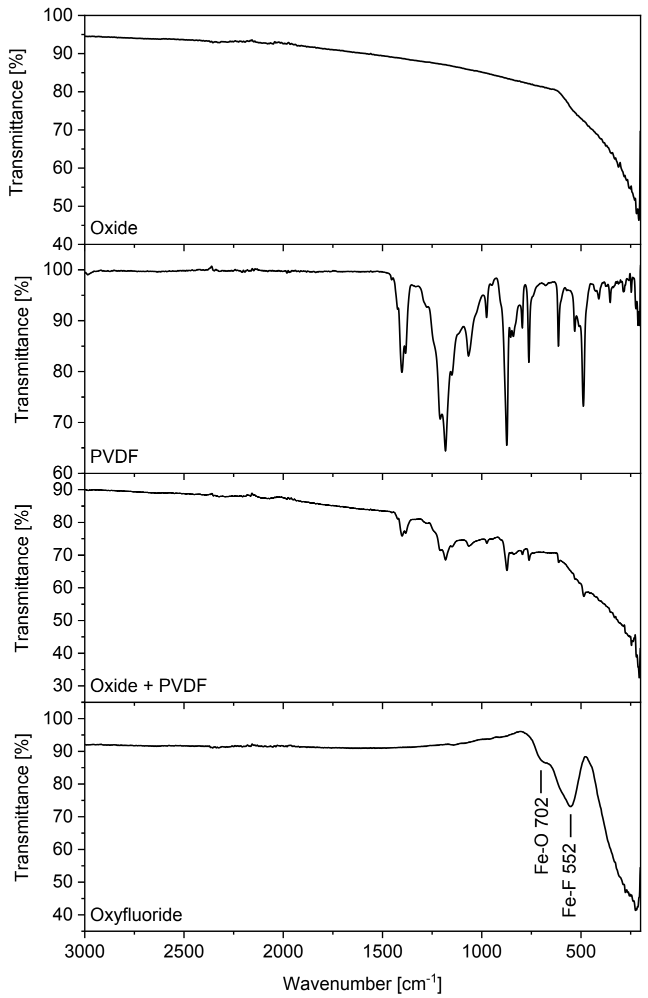

Figure S2: FT-IR-spectra of the precursor oxide with and without PVDF, pure PVDF and the resulting oxyfluoride. No residues of PVDF can be found in the final product.

Table S1: Selected atom distances for $La_{0.5}Sr_{3.5}Fe_3O_{7.5}F_{2.6}$ based on room temperature Rietveld refinement. The anionic positions are: O1 (te), O2 (te), O3 (ca), O4 (ce), F1 (ta), F2 (i).

Sr1/La1–…	Distance [Å]	Sr2/La2-…	Distance [Å]	Fe-…	Distance [Å]
O1	2.785(2)	F1	2.507(2)	Fe1-O3 (2x)	1.957(1)
O1	2.944(2)	F1	2.566(1)	Fe1-O4 (2x)	1.914(1)
O2	2.784(2)	F1	2.829(3)	Fe1-O4 (2x)	2.078(1)
O2	2.862(2)	F1	2.857(3)	Fe2-F1	2.494(1)
O3	2.606(2)	F2	2.397(6)	Fe2-O1	1.918(2)
O3	2.667(2)	F2	2.415(6)	Fe2-O1	1.995(2)
O3	2.889(2)	F2	2.576(6)	Fe2-O2	1.962(3)
O3	2.938(2)	F2	2.627(6)	Fe2-O2	2.026(2)
O4	2.452(2)	O1	2.471(2)	Fe2-O3	1.911(1)
O4	2.760(2)	O1	2.572(2)		
O4	2.941(2)	O2	2.558(2)		
		O2	2.718(2)		

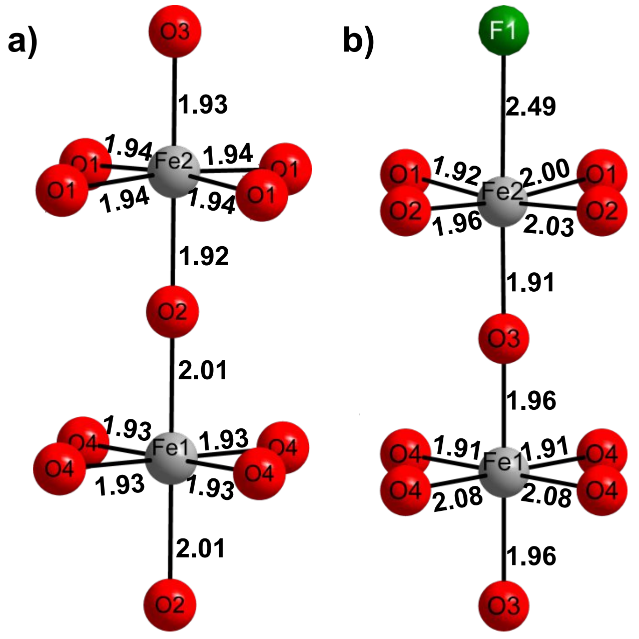

Figure S3: Comparison of the coordination geometries of the Fe-X-polyhedra for a) $La_{0.5}Sr_{3.5}Fe_3O_{8.75}$ and b) $La_{0.5}Sr_{3.5}Fe_3O_{7.5}F_{2.6}$. Atomic distances given in Å.

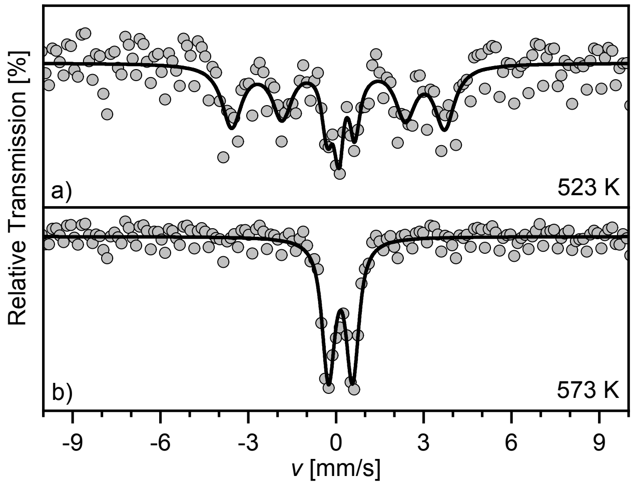

Figure S4: High-Temperature Mößbauer measurements of $La_{0.5}Sr_{3.5}Fe_3O_{7.5}F_{2.6}$ below (a) and above (b) magnetic ordering temperature.

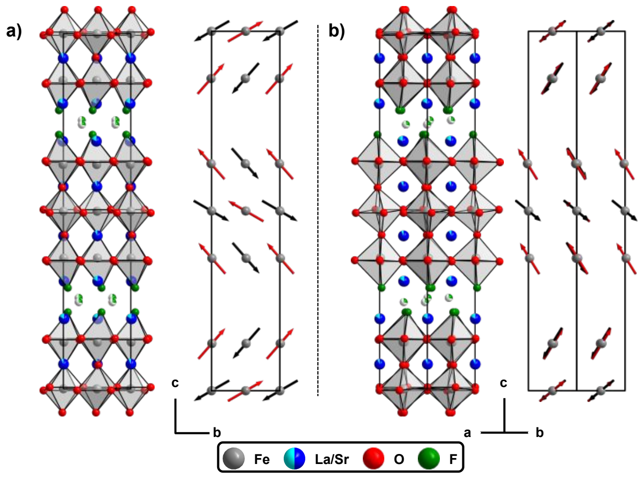

Figure S5: Crystal structure of $La_{0.5}Sr_{3.5}Fe_3O_{7.5}F_{2.6}$ with representation of Fe-coordination polyhedra (left) and orientation of the magnetic moments (right). a) Viewing direction parallel [100] b) parallel [110].

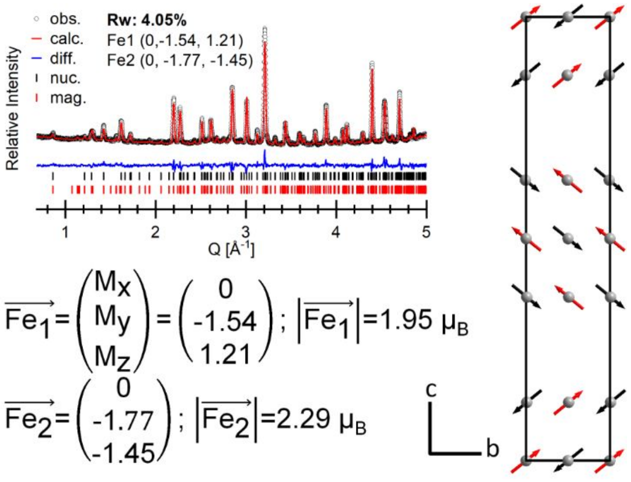

Figure S6: Top left: magnetic Rietveld refinement under inversion of the magnetic alignment parameters. Rw: 4.05 %. No significant changes compared to the reported refinement are found. Bottom left: Resulting magnetic vectors resp. moments. Right: Corresponding magnetic structure viewing direction parallel [100].
